# Supplementary material for: MetaRibo-Seq measures translation in microbiomes
Source: Nat Commun. 2020 Jun 29;11:3268. doi: 10.1038/s41467-020-17081-z (PMC7324362; doi:10.1038/s41467-020-17081-z)
Supplement: Supplementary file 10 — Supplementary Data 7 [file 41467_2020_17081_MOESM10_ESM.zip › File2/Confidence_VeryHigh_Taxonomy/115165_out.krona.html]

Javascript must be enabled to view this page.

members
magnitude
magnitudeUnassigned
count
unassigned
taxon
rank

115165\_out

4

2
superkingdom
3

1239
phylum
3

186801
class
3

order
186802
3

3
541000
family


SRS013476\_contig\_number\_32826SRS048870\_contig\_number\_12561
2
1952407
species

1
genus
1263

1638786
species

SRS019068\_contig\_number\_49423
1

1

SRS077194\_contig\_number\_contig-100\_14836.14836
